# Time-Course Effect of Electrical Stimulation on Nerve Regeneration of Diabetic Rats

**DOI:** 10.1371/journal.pone.0116711

**Published:** 2015-02-17

**Authors:** Yu-Ching Lin, Chia-Hong Kao, Chung-Chia Chen, Cherng-Jyh Ke, Chun-Hsu Yao, Yueh-Sheng Chen

**Affiliations:** 1 Lab of Biomaterials, School of Chinese Medicine, China Medical University, Taichung, Taiwan; 2 Department of Chinese Medicine, Taipei Medical University Hospital, Taipei, Taiwan; 3 Linsen (Chinese Medicine) Branch, Taipei City Hospital, Taipei, Taiwan; 4 Department of Orthopedics, College of Medicine, National Taiwan University, Taipei, Taiwan; 5 Department of Biomedical Informatics, Asia University, Wufeng District, Taichung, Taiwan; 6 Research Center for Chinese Medicine & Acupuncture, China Medical University, Taichung, Taiwan; Chang Gung University, TAIWAN

## Abstract

**Background:**

Electrical stimulation (ES) has been shown to promote nerve regeneration in rats with experimental diabetes induced using streptozotocin (STZ). However, the time-course effect of ES on nerve regeneration of diabetic animals has not been reported in previous studies. The present study attempted to examine the effect of different timing of ES after peripheral nerve transection in diabetic rats.

**Methodology/Findings:**

Fifty Sprague-Dawley rats were used in the study. They were classified into five groups. STZ-induced diabetes was created in groups A to D. Normal animals in group E were used as the non-diabetic controls. The sciatic nerve was transected and repaired using a silicone rubber conduit across a 10-mm gap in all groups. Groups A to C received ES for 15 minutes every other day for 2 weeks. Stimulation was initiated on day 1 following the nerve repair for group A, day 8 for group B, and day 15 for group C. The diabetic control group D and the normal control group E received no ES. At 30 days after surgery in group A, histological evaluations showed a higher success percentage of regeneration across the 10-mm nerve gap, and the electrophysiological results showed significantly larger mean values of evoked muscle action potential area and amplitude of the reinnervated gastrocnemius muscle compared with group D.

**Conclusions/Significance:**

It is concluded that an immediate onset of ES may improve the functional recovery of large nerve defect in diabetic animals.

## Introduction

Numerous strategies have been proposed for improving peripheral nerve regeneration [1–5]. Electrical stimulation (ES) is one of the beneficial methods for improving peripheral nerve regeneration not only in animal models but also in human clinical uses and has lots of research over the decades [6–13]. The application of ES immediately after crush nerve injury in rats may promote nerve regeneration and accelerate remyelination [6–8]. Evidence has proved that ES could lead to an elevated expression of brain derived neurotrophic factor (BDNF) in DRG sensory neurons [7–9] expression of calcitonin gene-related peptide (CGRP) and recruiting of macrophages [8]. Brief post-surgical low frequency electrical stimulation could accelerate axon regeneration and muscle reinnervation in carpal tunnel syndrome patients [10]. It has also demonstrated that ES can raise local blood flow to facilitate neurite extension and regeneration of transected nerve ends [14]. Additionally, electrical stimulation could accelerate the speed and improve the accuracy of motor axonal regeneration. Several neurotrophins such as BDNF, nerve growth factor (NGF), tyrosine kinase B (trkB) and C (trkC) receptors could induce cascade responses of motorneuron regeneration [15–18]. By the increase of BDNF and trkB receptor synthesis in motoneurons, ES could support motoneuronal survival and regeneration [15]. In these previous works, most researchers utilized ES to treat damaged nerves in normal rats. By comparison, nerve damage is more difficult to repair in diabetic animals since diabetes is a metabolic disorder with out-of-control blood sugar [19–21]. Due to sugar complexes attached on vessel walls, it could result in blood vessels narrowing down, and decreasing blood flow rate. This phenomenon leads to not only endoneurial hypoxia with concomitant energy deficit, but also ischemia and nerve dysfunction. Scientists have found that serious reduction of sciatic nerve blood flow could happen in streptozotocin (STZ)-induced diabetic rats [22, 23].

It has been confirmed that ES could be readily applicable as a supplementary treatment during nerve repair in the STZ-induced diabetic rats [24–26]. Additionally, it was found that the timing phenomenon is important for the effect of ES on nerve repair that a delayed ES could dramatically improve the recovery of regenerating nerves in normal rats [27]. Despite this knowledge, there is no experimental validation to know the time window effect of ES during nerve regeneration in an animal model with diabetes. Therefore, the aim of this study was to investigate the time-course effect of ES in STZ-induced diabetic rats on their nerve regeneration and functional recovery.

## Materials and Methods

### Induction of Diabetes

Prior to the beginning of the *in vivo* testing, the protocol was approved by the ethical committee for animal experiments of the China Medical University, Taichung, Taiwan. Diabetes was induced in adult male Sprague-Dawley rats (250–300 g, BioLasco Co, Ltd, Taipei, Taiwan) by tail vein injection of a single 50 mg/kg dose of STZ (Sigma Chemical Co, St Louis, MO). The STZ was solubilized in normal saline immediately before injection. Seven days after STZ injection, serum glucose measurements were determined on all animals with a glucose analyzer (Accu-Chek, Roche, Basel, Switzerland). Animals with an initial blood glucose of 300 mg/dl or greater qualified as diabetic. All animals became diabetic with a single injection of 50 mg/kg of STZ and were maintained in the high glucose levels during the whole course of the experiment.

### Implantation of Silicone Rubber Chambers

All rats were anesthetized with an inhalational technique (AErrane; Baxter, Deerfield, IL). The right sciatic nerves were severed into proximal and distal segments. The proximal stump was then secured with a single 9–0 nylon suture through the epineurium and the outer wall of a silicone rubber chamber (1.47 mm inner diameter, 1.96 mm outer diameter; Helix Medical, Inc, Carpinteria, CA). The distal stump was secured into the other end of the chamber. Both the proximal and the distal stumps were secured to a depth of 1 mm into the chamber, leaving a 10-mm gap between the stumps. The muscle and skin were closed. All animals were housed in temperature (22°C) and humidity (45%) controlled rooms with 12-hour light cycles. They had access to food and water *ad libitum*.

### ES Protocols

**Figure 1:**
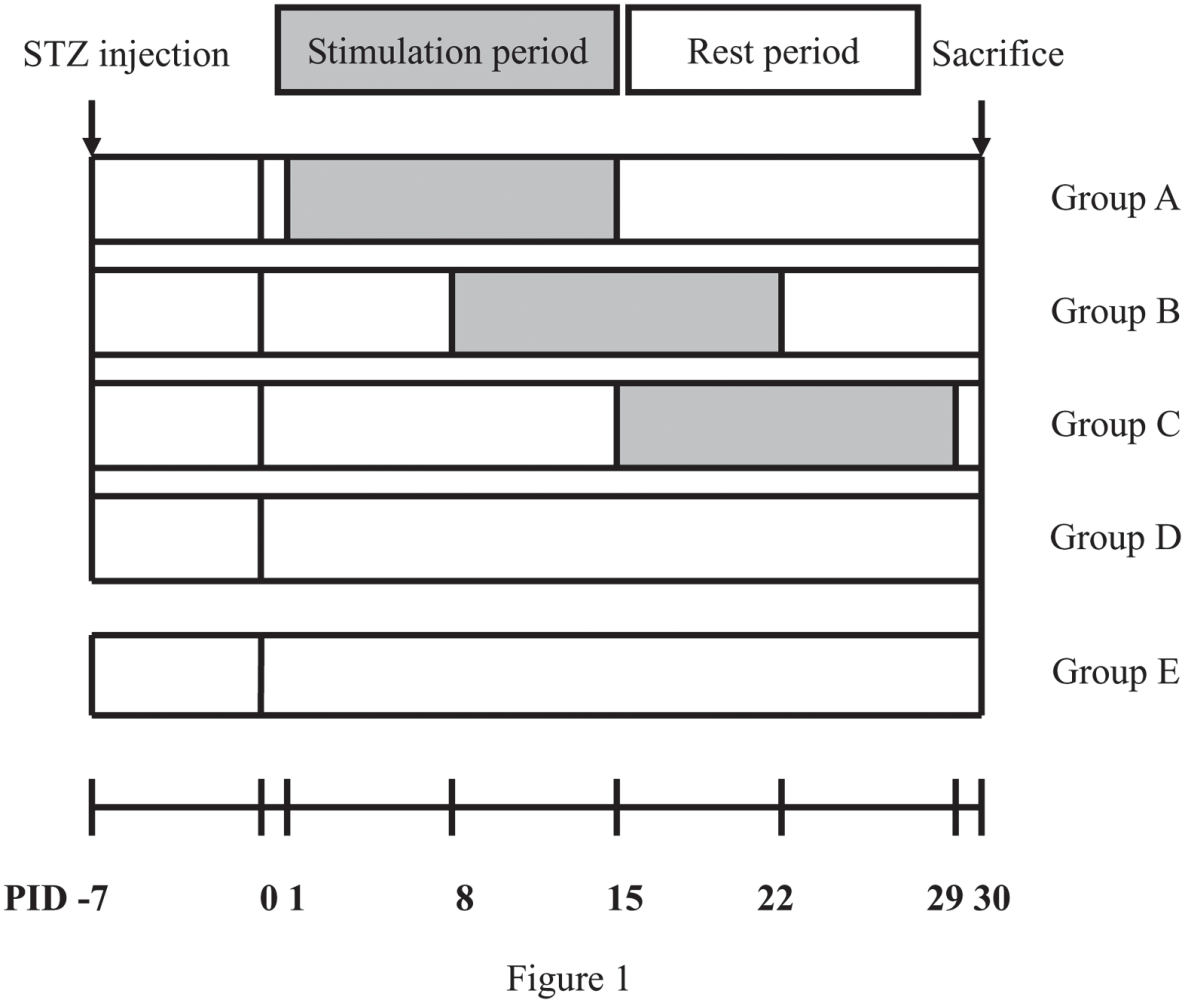
The time sequence of the study with stimulation periods as shaded blocks and rest periods as open blocks; induction of diabetes was performed at 7 days before the nerve surgery and all animals were sacrificed on PID 30. Abbreviation: PID, postinjury day.

The ES treatment protocol was previously reported [28] and the experimental flow is summarized in Fig. 1. In brief, animals were secured in a small cage and their stretched right leg and paw were held in place by rubber tapes. One stainless steel needle electrode (0.35 mm OD, 12 mm length) connected to the negative wick (cathode) of a stimulator (Trio 300; Ito, Tokyo, Japan) was inserted aseptically into the lateral aspect of the knee and the anode was positioned around the site of the hip joint. The positive and negative stimulating sites were near the proximal and distal ends of the implanted silicone tubes, respectively. The depth of insertion varied from 1 to 1.5 cm according to the thickness of skin and fatty tissues. The stimulation was applied to the animals at 2 Hz at the current intensity of 1 mA (pulse duration: 100 µs, square wave) to produce a visible muscle contraction for 15 minutes every other day from post-injury-day (PID) 1–15 (Group A, n = 10), 8–22 (Group B, n = 10) or 15–29 (Group C, n = 10). Group D (n = 10), diabetic controls, and Group E (n = 10), nondiabetic normal animals, underwent the nerve lesion and repaired with empty silicone rubber chambers. Both the groups D and E received no ES. Cutaneous blood flow in the hindlimb footpad ipsilateral to the injury of the rat in groups A-C was measured with a laser Doppler flowmetry device (wavelength, 780 nm; DRT4; Moor Instruments Ltd., Millwey, Axminster, UK) at the midpoint of the 15-day stimulation period. Similarly, cutaneous blood flows of the rat in groups D and E were measured at the mid time sequence (PID 15) of the study. All animals were anesthetized during the ES.

### Electrophysiological Techniques

On PID 30, all animals were re-anesthetized and the sciatic nerves were exposed and examined the success rate of cable formation in the silicone rubber conduits across the 10-mm gap. The nerve was given a supramaximal stimulus through a pair of needle electrodes placed directly on the sciatic nerve trunk, 5-mm proximal to the transection site. Latency, amplitude, and area of the evoked muscle action potentials (MAPs) were recorded from the gastrocnemius muscle with microneedle electrodes linked to a computer (Biopac Systems, Inc., Goleta, California). The latency was measured from stimulus to the takeoff of the first negative deflection. The amplitude and the area under the MAP curve from the baseline to the maximal negative peak were calculated. The MAP was then used to calculate the nerve conductive velocity (NCV), which was carried out by placing the recording electrodes in the gastrocnemius muscles and stimulating the sciatic nerve proximally and distally to the silicone rubber conduit. The NCV was then calculated by dividing the distance between the stimulating sites by the difference in latency time.

### Histological Techniques

Immediately after the recording of muscle action potential, all of the rats were perfused transacrdially with 150 ml normal saline followed by 300 ml 4% paraformaldehyde in 0.1 M phosphate buffer, pH 7.4. After perfusion, the L4 spinal cord and the distal stump outside the nerve gap were quickly removed and post-fixed in the same fixative for 3–4 h. Tissue samples were placed overnight in 30% sucrose for cryoprotection at 4°C, followed by embedding in optimal cutting temperature solution. Samples were the kept at -20°C until preparation of 18 μm sections was performed using a cryostat, with samples placed upon poly-L-lysine-coated slide. Immunohistochemistry of frozen sections was carried out using a two-step protocol according to the manufacturer’s instructions (Novolink Polymer Detection System, Novocastra). Briefly, frozen sections were required. Endogenous peroxidase activity was blocked with incubation of the slides in 0.3% H_2_O_2_, and nonspecific binding sites were blocked with Protein Block (RE7102; Novocastra). After serial incubation with rabbit- anti-CGRP polyclonal antibody 1:1000 (Calbiochem, Germany), Post Primary Block (RE7111; Novocastra), and secondary antibody (Novolink Polymer RE7112), the L4 spinal cord sections were developed in diaminobenzidine solution under a microscope and counterstained with hematoxylin. Similar protocols were applied in the sections from the distal stump except they were incubated with anti-rat CD68 1:100 to detect macrophages (AbD Serotec, Kidlington, UK). Sciatic nerve sections were taken from the middle regions of the regenerated nerve in the chamber. After the fixation, the nerve tissue was post-fixed in 0.5% osmium tetroxide, dehydrated, and embedded in Spurr’s resin. The tissue was then cut to 2 μm thickness by using a microtome (Leica EM UC6, Leica Biosystems, Mount Waverley, Australia) with a diamond knife, stained with toluidine blue.

### Image Analysis

All tissue samples were observed under an optical microscope (Olympus IX70; Olympus Optical Co, Ltd, Tokyo, Japan) with an image analyzer system (Image-Pro Lite; Media Cybernetics, Silver Spring, MD). CGRP-immunoreactivity (IR) in dorsal horn in the lumbar spinal cord was detected by immunohistochemistry as described previously [29]. The immuno-products were considered positive-labeled if their density level was over five times background levels. The area around central canal in the spinal cord was taken as a control area. Under a 400x magnification, the ratio of area occupied by positive CGRP-IR in dorsal horn ipsilateral to the injury following neurorrhaphy relative to the lumbar spinal cord was measured. The number of neural components in each nerve section was also counted. While counting the myelinated axons, at least 30 to 50 percent of the sciatic nerve section area randomly selected from each nerve specimen at a magnification of 400x was observed. The axon counts were extrapolated by using the area algorithm to estimate the total number of axons for each nerve [30]. Similarly, macrophages were counted in each nerve section at the distal stump and the density of macrophages was obtained by dividing the macrophage counts by the total nerve areas.

### Statistical Analysis

For the statistical analysis of immunohistochemical, morphometric, and electrophysiological measurements of regenerated nerves, data were collected by the same observer and expressed as mean ± standard deviation, and comparisons between groups were made by the 1-way analysis of variance (SAS 8.02). The Tukey test was then used as a post hoc test. Statistical significance was set at *P*< 0.05.

## Results

**Figure 2:**
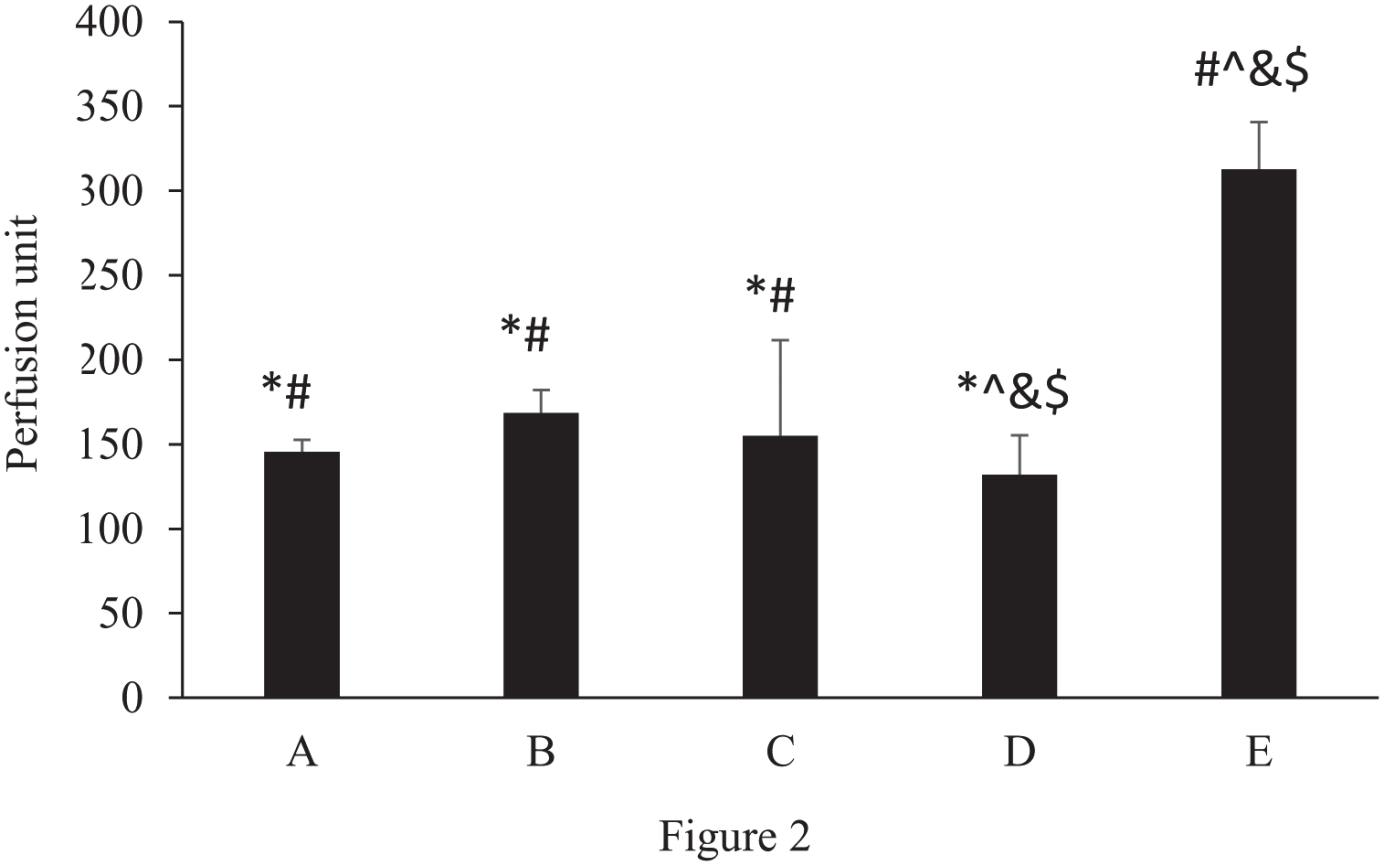
Blood perfusion to the ipsilateral hindpaw in response to ES applied at different timing in nondiabetic and diabetic rats (*p<0.05 compared to nondiabetic normal group, #*p*<0.05 compared to diabetic control group, &*p*<0.05 compared to group B, ^*p*<0.05 compared to group A).

The cutaneous blood flow in the ipsilateral hindpaw to the injury in response to ES applied at different periods after nerve repair in diabetic rats, the diabetic-controls, and the nondiabetic normal animals is shown in Fig. 2. Mean blood flow in nondiabetic normal animals measured was consistently about 300 perfusion units, which was significantly larger than that in the diabetic animals in groups A to D (p< 0.05; mean percentage increase: E vs. A = 115.1%, E vs. B = 85.6%, E vs. C = 101.7%, E vs. D = 136.9%). When brief ES was applied to the diabetic animals in groups A to C, their cutaneous blood flows were significantly enhanced as compared to the diabetic-controls (p< 0.05; mean percentage increase: A vs. D = 10.1%, B vs. D = 27.6%, C vs. D = 17.4%). There was no statistical difference between blood flow changes among the electrically stimulated diabetic animals in groups A to C.

**Figure 3:**
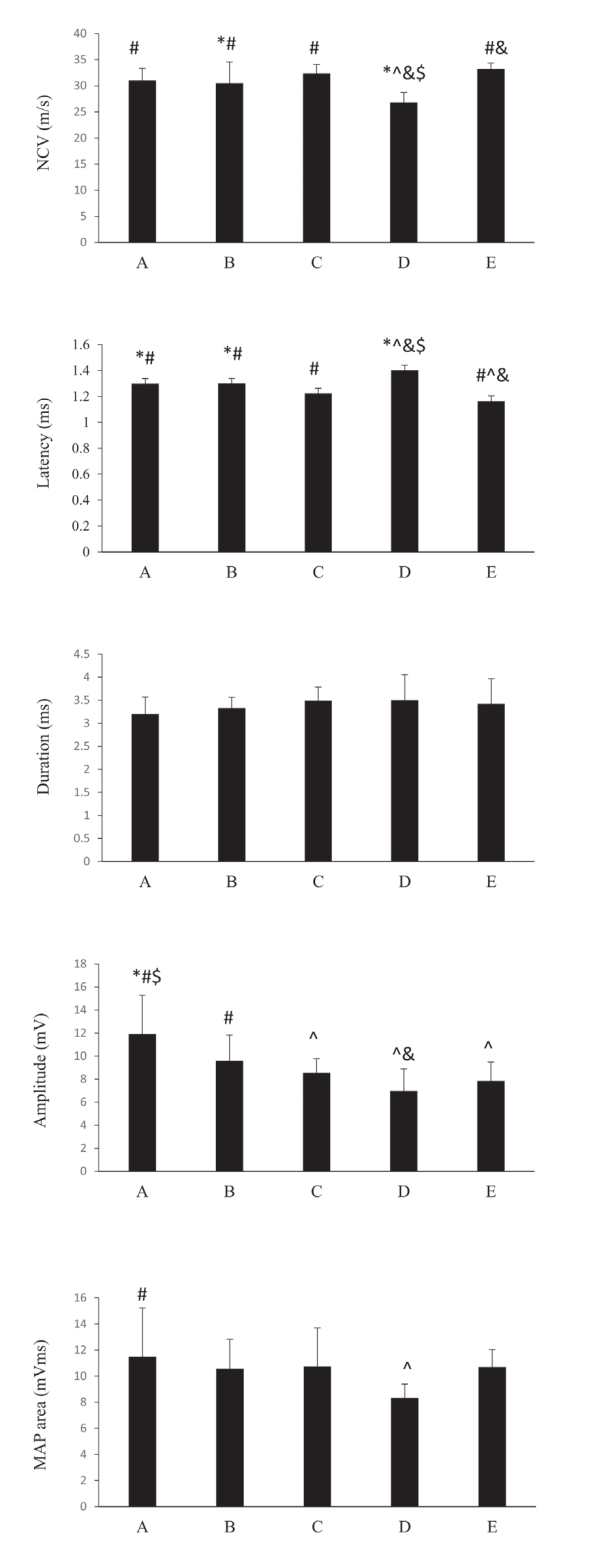
Electrophysiological measurements in response to ES applied at different timing in nondiabetic and diabetic rats (*p<0.05 compared to nondiabetic normal group, #*p*<0.05 compared to diabetic control group, $*p*<0.05 compared to group C, &*p*<0.05 compared to group B, ^*p*<0.05 compared to group A).

Electrophysiological data are shown in Fig. 3. It was noted that diabetic-controls had a significantly slower NCV as compared to all of the electrically stimulated diabetic animals and the nondiabetic animals (p < 0.05; mean percentage increase: A vs. D = 15.7%, B vs. D = 13.7%, C vs. D = 20.7%, E vs. D = 23.9%). No statistical difference in the NCV was seen among the ES-treated diabetic groups A to C. Similarly, the diabetic-controls had a significantly longer latency as compared to all of the ES-treated diabetic animals and the nondiabetic animals (p < 0.05; mean percentage increase: D vs. A = 7.8%, D vs. B = 7.8%, D vs. C = 14.5%, D vs. E = 20.7%). Constant data were seen in the duration measurements, resulting in no statistical difference among groups A to E. When diabetic animals were electrically stimulated, especially those in groups A and B, their amplitudes of MAP areas were significantly increased as compared to the diabetic-controls receiving no ES (p < 0.05; mean percentage increase: A vs. D = 70.8%, B vs. D = 37.4%). In addition, it was noted that ES application, beginning one day after nerve repair in group A, could significantly increase the MAP area as compared to the diabetic-controls (p < 0.05; mean percentage increase: A vs. D = 37.8%).

**Figure 4:**
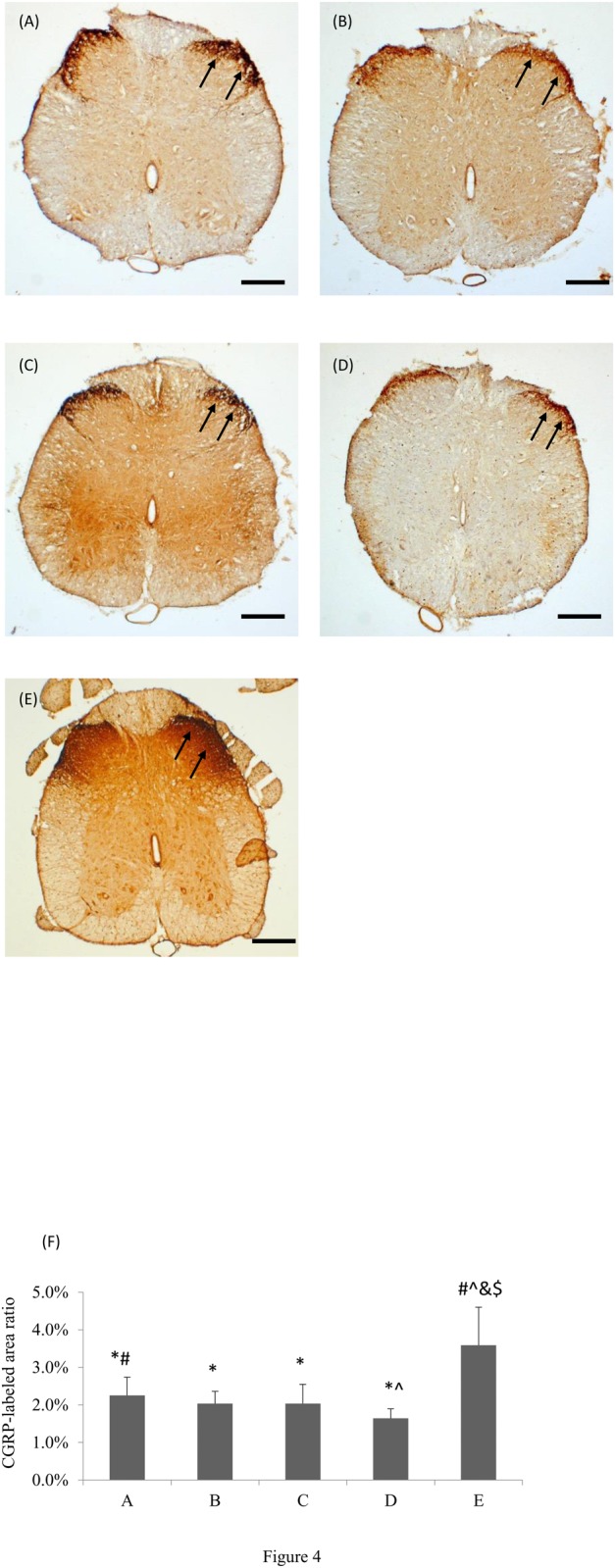
Photomicrographs demonstrating CGRP-IR (arrows) in laminae I-II of dorsal horn in the lumbar spinal cord after injury of sciatic nerves after ES treatment applied at different timing in diabetic rats from (A) group A (PID 1), (B) group B (PID 8), (C) group C (PID 15), (D) group D (no ES), and in nondiabetic normal rats from (E) group E (no ES). (F) Comparisons of CGRP-IR area ratios (*p<0.05 compared to nondiabetic normal group, #*p*<0.05 compared to diabetic control group, $*p*<0.05 compared to group C, &*p*<0.05 compared to group B, ^*p*<0.05 compared to group A). Each data represents the mean ± standard deviation. Bar = 200 µm.

Immunohistochemical staining showed that CGRP-labeled fibers were seen in the area of lamina III-V and lamina I-II regions in the dorsal horn ipsilateral to the injury in all of the rats (Fig. 4). Compared to the nondiabetic animals, the ratio of area occupied by positive CGRP-IR was significantly decreased in the diabetic animals even if they had received the ES treatment in groups A to C (p < 0.05; mean percentage decrease: A vs. E = 36.1%, B vs. E = 44.4%, C vs. E = 44.4%, D vs. E = 55.6%). However, it was noted that the positive CGRP-IR was significantly increased in group A as compared to the diabetic-controls (p < 0.05; mean percentage increase: A vs. D = 43.8%).

Success rate of cable formation in the silicone rubber conduits was measured. The successful cable formation was determined by bare eyes concerning there was continuous tissue extending between the proximal and distal nerve segments. A proportion (8 of 10 rats) formed regenerative outgrowth in the nondiabetic normal animals. In comparison, 90, 80, 70, and 60% showed regenerated nerve cables in the ES-treated diabetic groups of A to C and in the diabetic controls, respectively.

**Figure 5:**
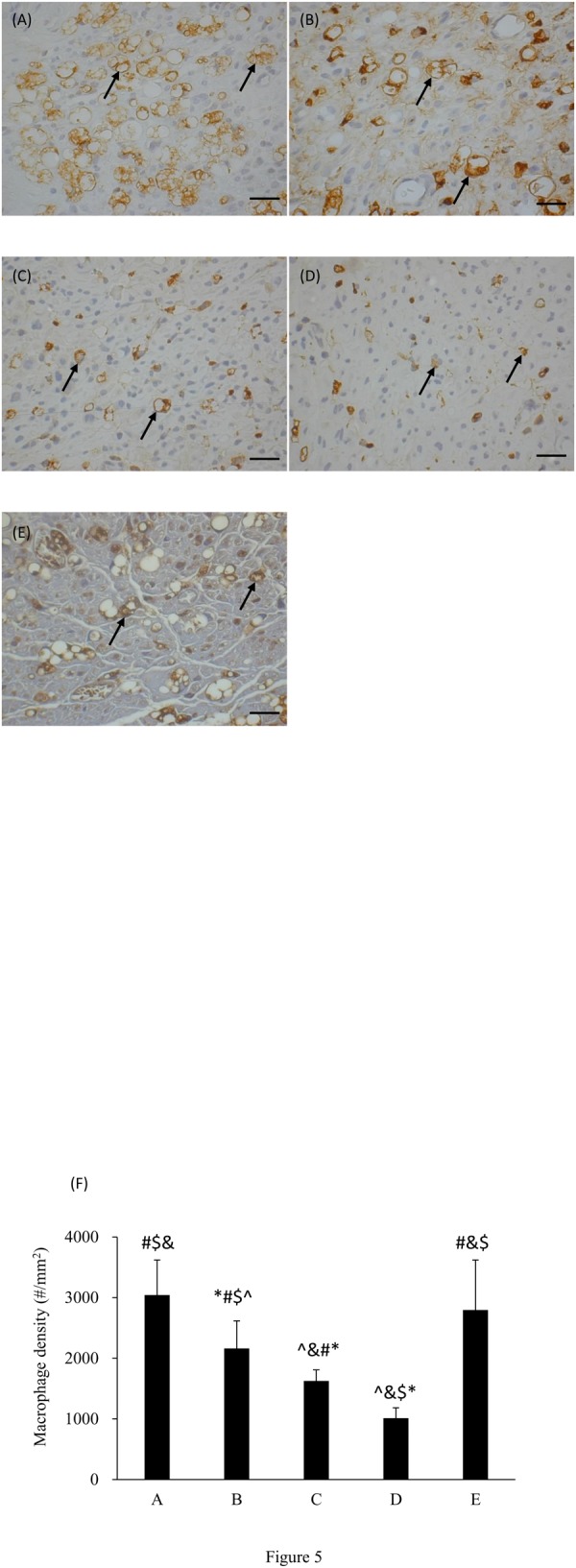
Photomicrographs demonstrating anti-rat CD68 immunoreactivity in macrophages (arrows) from cross sections of distal nerve cables after ES treatment applied at different timing in diabetic rats from (A) group A (PID 1), (B) group B (PID 8), (C) group C (PID 15), (D) group D (no ES), and in nondiabetic normal rats from (E) group E (no ES). (F) Macrophage density comparisons (*p<0.05 compared to nondiabetic normal group, #*p*<0.05 compared to diabetic control group, $*p*<0.05 compared to group C, &*p*<0.05 compared to group B, ^*p*<0.05 compared to group A). Each data represents the mean ± standard deviation. Bar = 20 µm.

A significantly reduced number of macrophages were observed in the diabetic controls as compared to the nondiabetic normal animals (p < 0.05; mean percentage decrease: D vs. E = 63.8%). It was noted that no matter when ES was applied to the diabetic animals, macrophages were significantly recruited back into the nerve stumps (Fig. 5). The density of macrophages in group A, receiving ES beginning one day after nerve repair in diabetic animals, even reached a 8% increase more than the nondiabetic normal animals (p > 0.05).

**Figure 6:**
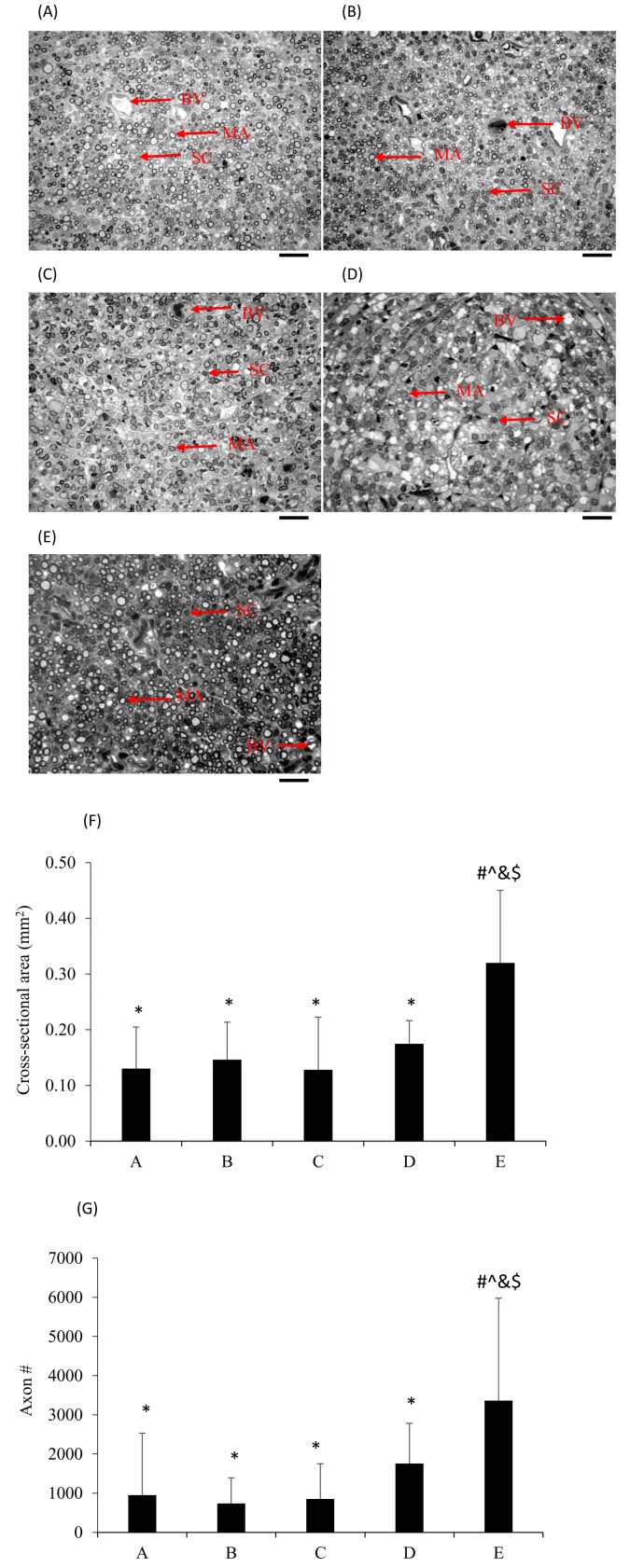
Images of regenerative nerves in transverse section after ES treatment applied at different timing in diabetic rats from (A) group A (PID 1), (B) group B (PID 8), (C) group C (PID 15), (D) group D (no ES), and in nondiabetic normal rats from (E) group E (no ES). Nerves show the typical aspect of a regenerated nerve, with myelinated axons (MA), Schwann cells (SC) and blood vessels (BV), which tended to disperse in the endoneurium. (F) Comparisons of cross-sectional area and (G) axon numbers (*p<0.05 compared to nondiabetic normal group, #*p*<0.05 compared to diabetic control group, $*p*<0.05 compared to group C, &*p*<0.05 compared to group B, ^*p*<0.05 compared to group A). Each data represents the mean ± standard deviation. Bar = 20µm.

Semi-thin transverse sections of the middle regions of the successfully regenerated sciatic nerves were used to observe their morphological changes. The regenerated nerves had a symmetric rim surrounding a cellular core. Using toluidine blue stain, Schwann cells and fibroblast nucleus as well as blood vessels were stained darkly. Regenerated axons surrounded by crescent-shaped Schwann cells in the nondiabetic-rat nerves were compacted with larger diameters than diabetic-rat nerves (Fig. 6). Morphometric analysis showed that endoneurial spaces and myelinated axon counts were significantly decreased in the diabetic animals, no matter whether they received the ES or not, versus those of nondiabetic normal animals (p < 0.05; mean percentage decrease: A vs. E = 71.7%, B vs. E = 78.1%, C vs. E = 74.7%, D vs. E = 47.8%). By comparison, there was no significant difference between the electrically stimulated diabetic animals in groups A to C and the diabetic-controls for their morphometric data (p > 0.05).

## Discussion

The capacity of nerve regeneration has been reported dramatically declined in diabetic animals [31–33]. Therefore, the treatment of diabetic nerve repair has been a challenge for clinicians. The present study investigated the effect of ES applied at different timing after repair on nerve regeneration and functional recovery in diabetic rats. Brief ES was capable of enhancing cutaneous blood flow in diabetic animals. We also found that stimulated regenerating nerves, especially initiated at Day 1 following nerve repair had significantly larger amplitudes and MAP areas as compared to the diabetic-controls with no ES. Also, the expression of CGRP and the recruiting of macrophages were significantly up-regulated in the diabetic animals receiving ES from PID 1. In addition, the same ES-treated diabetic animals from PID 1 had the highest success rate of cable regeneration in bridging conduits, up to 90% compared to only 60% in the untreated diabetic-controls. All these findings highlight the possibility of using early ES as a useful tool to accelerate nerve regeneration and functional recovery in diabetic animals.

In our previous work, it was found that a short delay in the onset of ES is beneficial to the recovery of transection injury in normal rats [27]. Delayed ES for 8 days produced better regeneration, approximately a two-fold increase in myelinated axons that grew across the 10-mm gap, more regenerated blood vessels and larger nerve areas than stimulation at 1 day in transection injury in normal rats. In the present study, however, no such delayed beneficial effects were found. Instead, an immediate ES applied at PID 1 could exert the most profound effect on nerve regenerative ability in diabetic rats. These findings showed that the time-course of ES is an important factor for the final recovery after peripheral nerve injuries in the diabetic animal model. The mechanism underlying the accelerated nerve recovery by early ES in diabetic rats is still uncertain. A possible explanation is that the early ES effect on diabetic nerve regeneration could depend on improvements in nerve perfusion. It has been reported that regional ischemia of arteries of the limb is commonly seen in diabetics, which could impair regeneration and repair in diabetic nerves [34]. In the present study, it was found that brief ES could induce a transient rise in skin perfusion in diabetic rats, supporting their metabolic requirements of regenerative processes. In addition, rises in local blood flow induced by early ES may help to deliver more injury-related inflammatory substances to the injury site to accelerate inflammatory repair response [35].

Functional recovery is generally poor for diabetic nerve trunk injuries. Thus far, many studies have been performed to identify the mechanisms for poor functional performance in diabetics after nerve repair, such as peripheral neuropathy [36] and ischemic microvascular angiopathy resulting from organ and systemic complications of diabetes mellitus [37]. In the present study, it was found that early ES application beginning one day after nerve repair could significantly increase the MAP area and the amplitude of the reinnervated gastrocnemius muscle as compared to the diabetic-controls. These results indicated that target muscles can be reinnervated in the short term using the ES technique. Besides, the therapeutic time window of ES for the regeneration and reinnervation to occur in the diabetic peripheral nervous system is critical and the ES should be applied as early as possible after the muscle denervation.

Peripheral diabetic neuropathy is one of the serious complications of diabetes, which may impair peripheral nerve regeneration. Structural changes in peripheral blood vessels and reductions in blood flow, decreases in action of vasoactive neuropeptides such as CGRP to render an ischemic environment in nerve microvessels following nerve injury, delays in recruitment of macrophages to clean degrading myelin in injured peripheral nerve fibers, and reductions in neurotrophic supports for regenerating nerve tissues all could be the causes for failed regeneration and impaired myelinated axon population in diabetic peripheral nerves [38–41].

CGRP is a potent vasodilator and neuropeptide that may participate in the injury response of peripheral nerve [42]. It may regulate terminal sprouting by increasing synthesis in axotomized motor neurons [43]. After a peripheral nerve transection in the adult animal, there is a robust upregulation of CGRP messenger RNA (mRNA) together with mRNAs encoding cytoskeletal and growth-related proteins [44]. Furthermore, after CGRP mRNA expression upregulated, there is an increase of CGRP-IR fibers in the dorsal horn ipsilateral to the injury [29]. In the present study, it was noted that the positive CGRP-IR was significantly increased in diabetic animals treated with early ES beginning at PID 1 as compared to the diabetic-controls. By comparison, the CGRP content in laminae of the spinal dorsal horn in the diabetic rats was dramatically declined at later time points of ES after nerve repair. However, the rises in cutaneous blood flow at all time points of ES after nerve repair seen in the present study were similar. Thus, the CGRP accumulation in a time-related fashion could combine with other possible actions to induce equal local vasodilation.

Poor macrophage infiltration following nerve injury is commonly seen in diabetic animals, which may delay Wallerian degeneration resulting in impairment in regeneration [45]. It has been demonstrated that the regenerative response of diabetic nerves can be improved through treatment of ES by activating the macrophages appropriately infiltrate the endoneurium [24, 25]. Similarly seen in the present study, the diabetic rats treated with early ES had dramatically improved macrophage invasion, which even caught up to the normal control regenerative end point. Since macrophages can create an environment favorable to axonal regeneration [46], the optimal timing of ES treatment for diabetic animals to recruit more macrophages is suggested as early as 1 day after nerve repair.

The morphometric analysis seen in the present study could not show the early ES beneficial effects on regenerating diabetic nerves. No significant difference was observed between the ES-treated diabetic animals and the diabetic-controls. Although ES could accelerate the formation of nerve cables connecting the dissected stumps, promote release of positive CGRP-IR to increase local blood flow, restore recruitment of macrophages, these did not significantly impact the morphology of the regenerated diabetic nerves. It implied that morphometry, as observed in this study, could not provide information on the improvement of diabetic nerve function by ES. The lack of a direct relationship between morphometric data and functional studies in animals has also been reported by others. de Medinaceli concluded that morphometric analysis following traumatic dissection could provide evidence realizing the changes in regenerating nerves but gave no clues as to their functional recovery [47]. Only in the case of individual axons or very mild nerve injury does function restoration closely match morphological findings. Brenner et al. also indicated that histologic differences only could be seen at early stages of nerve regeneration and functional differences at later stages [48]. These results implied that selection of appropriate time points to evaluate regenerated nerves is a critical consideration.

## Conclusion

Our findings substantiate the conclusion that the time-course of ES is of importance for the final recovery after peripheral nerve injuries. The use of an immediate ES paradigm could accelerate the recruitment of macrophages, enhance the expression of CGRP that nerve regeneration required and improve functional restoration. Those are important for successful nerve regeneration in diabetic animals.
